# Gap in Sexual Dysfunction Management Between Male and Female Patients Seen in Primary Care: An Observational Study

**DOI:** 10.1007/s11606-024-09004-1

**Published:** 2024-09-04

**Authors:** Elizabeth E. Stanley, Elizabeth Pfoh, Laura Lipold, Kathryn Martinez

**Affiliations:** 1 Cleveland Clinic Lerner College of Medicine, Cleveland, USA; 2 Cleveland Clinic Center for Value-Based Care Research, Cleveland, OH USA; 3 Department of Family Medicine, Cleveland Clinic, Cleveland, OH USA

**Keywords:** female sexual dysfunction, genitourinary syndrome of menopause, erectile dysfunction, women’s health, flibanserin, bremelanotide

## Abstract

**Background:**

Female sexual dysfunction (FSD), defined as clinically distressing problems with desire, arousal, orgasm, or pain, affects 12% of US women. Despite availability of medications for FSD, primary care physicians (PCPs) report feeling underprepared to manage it. In contrast, erectile dysfunction (ED) is frequently treated in primary care.

**Objective:**

To describe differences in patterns of FSD and ED diagnosis and management in primary care patients.

**Design:**

Retrospective observational study.

**Subjects:**

Primary care patients with an incident diagnosis of FSD or ED seen at a large, integrated health system between 2016 and 2022.

**Main Measures:**

Sexual dysfunction management (referral or prescription of a guideline-concordant medication within 3 days of diagnosis), patient characteristics (age, race, insurance type, marital status), and specialty of physician who diagnosed sexual dysfunction. We estimated the odds of FSD and ED management using mixed effects logistic regression in separate models.

**Key Results:**

The sample included 6540 female patients newly diagnosed with FSD and 16,591 male patients newly diagnosed with ED. Twenty-two percent of FSD diagnoses were made by PCPs, and 38% by OB/GYNs. Forty percent of ED diagnoses were made by PCPs and 20% by urologists. Patients with FSD were managed less frequently (33%) than ED patients (41%). The majority of FSD and ED patients who were managed received a medication (96% and 97%, respectively). In the multivariable models, compared to diagnosis by a specialist, diagnosis by a PCP was associated with lower odds of management for FSD patients (aOR, 0.59; 95% CI, 0.51–0.69) and higher odds of management (aOR, 1.52; 95% CI, 1.36–1.64) for ED patients.

**Conclusions:**

Primary care patients with FSD are less likely to receive management if they are diagnosed by a PCP than by an OB/GYN. The opposite was true of ED patients, exposing a gap in the quality of care female patients receive.

**Graphical abstract:**

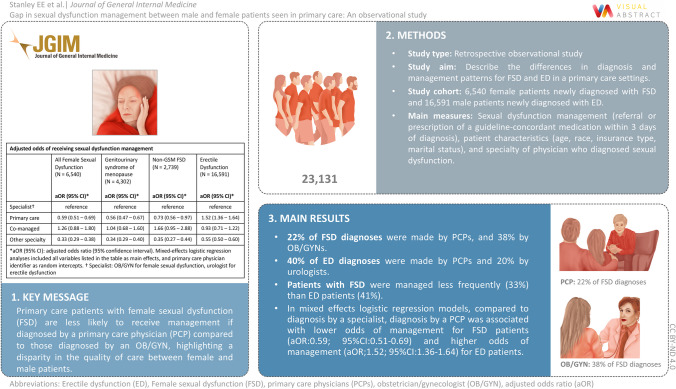

**Supplementary Information:**

The online version contains supplementary material available at 10.1007/s11606-024-09004-1.

## INTRODUCTION

Female sexual dysfunction (FSD) is an umbrella term for problems with women’s sexual function (i.e., problems with desire, arousal, orgasm, or pain) so bothersome they cause clinically significant distress.^1^ Survey studies indicate that 12% of women experience distressing problems with desire, arousal, or orgasm^2^ and 13–21% of women experience painful sex.^3,4^ While common, FSD is thought to be under-diagnosed and under-treated. In contrast, erectile dysfunction (ED), which affects approximately 20% of men,^5,6^ is frequently treated in primary care.^7^

The past decade has brought advances in options for and guidance about FSD treatment. Several professional societies published guidelines regarding the diagnosis and treatment of FSD.^1,8,9^ Further, there are a growing number of sub-specialists focused on treatment of sexual dysfunction to whom patients can be referred.^10^ Most notably, the FDA approved novel drugs, flibanserin and bremelanotide, for treatment of hypoactive sexual desire disorder.^11,12^ Women using flibanserin, a daily pill, report more satisfying sexual encounters per month versus those using placebo.^12^ Women using bremelanotide, an injection used prior to intercourse, reported satisfaction with 66% of sexual encounters, compared to 40% among placebo users.^13^ Bremelanotide’s efficacy is similar to that of phosphodiesterase 5 inhibitors (PDE5is) for erectile dysfunction (ED), as PDE5is result in successful sexual intercourse in 69% of attempts, compared to 36% among placebo users.^14^ However, in contrast to PDE5is, prescriptions of flibanserin and bremelanotide are thought to remain low.

Prior to the availability of these FDA-approved treatments, a survey of primary care physicians (PCPs) found 90% had never diagnosed hypoactive sexual desire disorder and 98% had never prescribed a medication for it.^15^ In comparison, a survey of American obstetrician/gynecologists (OB/GYNs) found 40% reported routinely asking patients about sexual function.^16^ While this points to potentially greater experience addressing FSD among OB/GYNs, these studies also suggest that FSD is likely under-diagnosed and under-treated by both specialties. The rate of medication prescription for ED in primary care is much higher. One study found PCPs prescribed medication at 67% of encounters where ED was included on the problem list.^7^

A study characterizing the extent of the purported underdiagnosis and undertreatment of FSD at the health-system level is needed. We determined the frequency of sexual dysfunction diagnoses and management for female and male patients and their association with patient and physician factors in a large, integrated health system. Then, we assessed the correlation between propensity to manage sexual dysfunction among female versus male patients among individual primary care physicians.

## METHODS

This retrospective study uses data from the electronic health record (EHR) of the Cleveland Clinic Health System (CCHS). We included adults (age ≥ 18) who saw a primary care (family or internal medicine) physician in Ohio between December 2016 and December 2022. We excluded transgender patients and those with a history of gynecologic or breast cancer, given differing clinical care needs.^17^ This study was approved by Cleveland Clinic’s Institutional Review Board.

### Sexual Dysfunction Diagnosis

We identified diagnosis of FSD or ED using ICD codes (Supplemental Table 1) and patient sex (e.g., female for FSD and male for ED). We included the following FSD diagnoses: female sexual interest/arousal disorder (FSIAD), female orgasm disorder, genitopelvic pain/penetration dysfunction (GPPD, i.e., dyspareunia, vulvodynia, vaginismus), genitourinary syndrome of menopause (GSM), and unspecified female dysfunction. For male patients, we included diagnosis of erectile dysfunction. We retrieved all instances of these ICD codes in each patient’s record. When determining whether patients were newly diagnosed during the study period, we required that they had no ICD code for that condition recorded prior to the study period.

### Sexual Dysfunction Management

We defined management of sexual dysfunction as receiving an appropriate medication prescription or referral within 3 days following the index sexual dysfunction diagnosis. We defined appropriate prescriptions and referrals according to recent guidelines from the International Society for the Study of Women’s Sexual Health (ISSWSH), American College of Obstetrics & Gynecology (ACOG), and the American Academy of Family Medicine (AAFP).^1,8,9,18,19^ Treatment for FSD can be complex due to the overlapping nature of conditions and lack of a single, best first-line treatment for any one condition. We therefore broadly defined possible management strategies with the aim of capturing all possible types of management. We included all first-line medications mentioned in these guidelines, even in the case of conflicting recommendations or limited evidence to support a treatment.

We considered a referral to OB/GYN or the women’s health specialty clinic appropriate management for all FSD diagnoses, referral to psychiatry or psychology appropriate management for all FSD diagnoses excluding GSM, referral to pelvic floor physical therapy appropriate management for GPPD, and referral to urology appropriate management for ED.

We considered flibanserin, bremelanotide, bupropion, transdermal testosterone, vaginal lubricants, or PDE5is to be appropriate medications for FSIAD.^1,8,9^ We considered vaginal estrogen, vaginal DHEA, vaginal lubricants, and ospemifene to be appropriate medications for GSM.^1,8^ We considered muscle relaxants (diazepam, cyclobenzaprine, orphenadrine, methocarbamol, metaxalone, baclofen, tizanidine, chlorzoxazone, carisoprodol), gabapentin, topical lidocaine, topical capsaicin, and treatments for GSM to be appropriate medications for GPPD management.^1,18^ We considered prescription of a PDE5i an appropriate treatment for ED.^19^

### Patient Characteristics

We retrieved patient characteristics (age, race, marital status, and insurance status) from the EHR. We used each patient’s age and insurance status at the time of their first sexual dysfunction diagnosis. We categorized patients by the specialty of clinician who diagnosed their sexual dysfunction. We considered patients diagnosed by a PCP if they had a PCP visit in the 7 days prior to the first date a sexual dysfunction diagnosis was noted in their chart. We considered patients diagnosed by a specialist (OB/GYN for FSD and urology for ED) if they had a specialist visit in the 7 days prior to the first date a sexual dysfunction diagnosis was noted in their chart. We considered patients to be co-managed if they had both a PCP and specialist visit in the 7 days preceding their first diagnosis. We categorized patients with neither a specialist nor PCP visit in the 7 days preceding their initial diagnosis as diagnosed by “other” clinician type (such as oncologist or psychiatrist).

### Statistical Analyses

We calculated the proportion of patients ever diagnosed with FSD and ED, and among those diagnosed during the study period, we described their demographic characteristics, as well as type of management received. We used chi-squared tests to compare between ED and FSD patients the distribution of diagnosing providers’ specialty and proportion receiving management. We compared characteristics between those receiving management and those not using chi-squared tests.

We stratified the population by sex. We then ran mixed-effects logistic regression models, clustering by patients’ primary care physician, to estimate differences in odds of receiving appropriate management at diagnosis. Given more established recommendations regarding treatment of GSM, we also ran regression models among female patients diagnosed with GSM and female patients with any other type of FSD. Models included patient age, race, insurance type, and diagnosing specialty (PCP, specialist, co-managed, or other). As one FSD treatment, bremelanotide, was approved partway through the study period (2019), we conducted a sensitivity analysis including only patients diagnosed with sexual dysfunction after 2019.

Since we defined ED management as either prescription of a PDE5i or referral to a urologist and some patients were initially diagnosed by urologists (making referral to another urologist less likely), we conducted a sensitivity analysis wherein we defined ED management as only a PDE5i prescription.

For each PCP, we calculated the proportion of patients they diagnosed who received management for ED and FSD. We calculated the Pearson’s correlation coefficient between individual physicians’ FSD and ED management rates. Analyses were conducted in R version 4.3.2.

## RESULTS

### Sample Characteristics

Among the 321,782 male and 369,647 female study-eligible patients seen by a PCP during the study period, 4% (*N* = 13,857) of female patients were ever diagnosed with FSD and 10% (*N* = 31,789) of male patients were ever diagnosed with ED. Two percent (*N* = 6540) of patients were newly diagnosed with FSD during the study period and 5.2% (*N* = 16,591) with ED, which is our analytic sample. Among patients with an FSD diagnosis during the study period, GSM was the most common (66%). The majority of patients with a female sexual dysfunction diagnosis during the study period were White (85%), post-menopausal (59%), married (66%), and had commercial insurance (54%) (Table 1). The majority of patients with an erectile dysfunction diagnosis during the study period were White (81%) and married (70%).

**Table 1:** Characteristics of Patients Newly Diagnosed with Sexual Dysfunction, Overall and By Management Status

	Female sexual dysfunction			Erectile dysfunction		
	Overall	Not managed	Managed*	Overall	Not managed	Managed*
*N* (row %)	6540 (100)	4388 (67)	2152 (33)	16,591 (100)	9835 (59)	6756 (41)
Age: mean (sd)	57 (15.3)	56 (16.4)	60 (12.0)	61 (12.0)	62 (12.3)	59 (11.6)
Race: *N* (%)						
White	5575 (85.2)	3730 (85.0)	1845 (85.7)	13,401 (80.8)	8014 (81.5)	5387 (79.7)
Black	583 (8.9)	391 (8.9)	192 (8.9)	2309 (13.9)	1335 (13.6)	974 (14.4)
Multiracial	146 (2.2)	106 (2.4)	40 (1.9)	376 (2.3)	209 (2.1)	167 (2.5)
Asian/Pacific Islander	107 (1.6)	76 (1.7)	31 (1.4)	175 (1.1)	94 (1.0)	81 (1.2)
Other/unknown/declined	129 (2.0)	85 (1.9)	44 (2.0)	330 (2.0)	183 (1.9)	147 (2.2)
Marital status: *N* (%)						
Married/domestic partner	4303 (65.8)	2831 (64.5)	1472 (68.4)	11,574 (69.8)	6867 (69.8)	4707 (69.7)
Legally separated/divorced/widowed	1187 (18.1)	779 (17.8)	408 (19.0)	2220 (13.4)	1331 (13.5)	889 (13.2)
Single	1002 (15.3)	749 (17.1)	253 (11.8)	2671 (16.1)	1558 (15.8)	1113 (16.5)
Unknown/other	48 (0.7)	29 (0.7)	19 (0.9)	126 (0.8)	79 (0.8)	47 (0.7)
Insurance: *N* (%)						
Commercial	3531 (54.0)	2365 (53.9)	1166 (54.2)	7791 (47.0)	4213 (42.8)	3578 (53.0)
Medicaid	652 (10.0)	478 (10.9)	174 (8.1)	1541 (9.3)	923 (9.4)	618 (9.1)
Other/Self	133 (2.0)	91 (2.1)	42 (1.9)	367 (2.2)	191 (1.9)	176 (2.6)
Medicare	2224 (34.0)	1454 (33.1)	770 (35.8)	6892 (41.5)	4508 (45.8)	2384 (35.3)
Diagnosing provider: *N* (%)						
Specialist^‡^	2507 (38.3)	1440 (32.8)	1067 (49.6)	3344 (20.2)	1977 (20.1)	1367 (20.2)
PCP	1445 (22.1)	949 (21.6)	496 (23.0)	6600 (39.8)	3132 (31.8)	3468 (51.3)
Co-managed	133 (2.0)	66 (1.5)	67 (3.1)	246 (1.5)	149 (1.5)	97 (1.4)
Other specialty	2455 (37.5)	1933 (44.1)	522 (24.3)	6401 (38.6)	4577 (46.5)	1824 (27.0)
Diagnosis: *N* (%)						
Genitourinary syndrome of menopause	4302 (65.8)	2524 (57.5)	1778 (82.6)	NA	NA	NA
Genitopelvic pain/penetration dysfunction	1878 (28.7)	1482 (33.8)	396 (18.4)	NA	NA	NA
Female sexual interest/arousal disorder	944 (14.4)	685 (15.6)	259 (12)	NA	NA	NA
Female orgasm disorder	66 (1)	49 (1.1)	17 (0.8)	NA	NA	NA
Unspecified FSD	52 (0.8)	37 (0.8)	15 (0.7)	NA	NA	NA
Received referral: *N* (%)	159 (2.4)	0 (0)	159 (7.4)	295 (1.8)	0 (0)	295 (4.4)
Received prescription: *N* (%)	2061 (31.5)	0 (0)	2061 (95.8)	6565 (39.6)	0 (0)	6633 (98.2)
Vaginal estrogen	1749 (26.7)	0 (0)	1749 (39.9)	NA	NA	NA
Flibanserin	135 (2.1)	0 (0)	135 (3.1)	NA	NA	NA
Vaginal DHEA	71 (1.1)	0 (0)	71 (1.6)	NA	NA	NA
Bupropion	58 (0.9)	0 (0)	58 (1.3)	NA	NA	NA
Muscle relaxant	45 (0.7)	0 (0)	45 (1)	NA	NA	NA
Gabapentin	31 (0.5)	0 (0)	31 (0.7)	NA	NA	NA
Vaginal lubricant	15 (0.2)	0 (0)	15 (0.3)	NA	NA	NA
Bremelanotide	10 (0.2)	0 (0)	10 (0.2)	NA	NA	NA
Ospemifene	3 (0)	0 (0)	3 (0)	NA	NA	NA
PDE5i^§^	2 (0)	0 (0)	2 (0)	6565 (39.6)	0 (0)	6565 (97.2)
Topical lidocaine	1 (0)	0 (0)	1 (0)	NA	NA	NA
Transdermal testosterone	1 (0)	0 (0)	1 (0)	NA	NA	NA
Topical capsaicin	0 (0)	0 (0)	0 (0)	NA	NA	NA

^*^Management is defined as receiving either a guideline-concordant medication or referral within 3 days of a diagnosis being made

^‡^Specialist: OB/GYN for female sexual dysfunction, urologist for erectile dysfunction

^§^*PDE5i*, phosphodiesterase 5 inhibitor

There were significant differences in the proportion of FSD and ED patients diagnosed by clinician type (*p* < 0.001). FSD patients were less frequently diagnosed by a PCP (22%) compared to ED patients (40%, Table 1). FSD was more frequently diagnosed by an OB/GYN than a PCP (38% vs. 22%). ED was more frequently diagnosed by a PCP (40% vs. 20%) than by a urologist.

### Management of Sexual Dysfunction

Fewer FSD patients were managed (33%) than ED patients (41%, *p* < 0.001). Among patients with FSD, 2061 (32%) were prescribed a medication and 159 (2.4%) received a referral. Patients with GSM were more likely to receive management compared to patients with other FSD diagnoses (41% vs. 21%, *p* < 0.001). Among patients with any FSD diagnosis, vaginal estrogen was the most frequently prescribed medication (27% of patients, Table 1). Flibanserin was the second most frequently prescribed: 14% of FSIAD patients received a prescription. Bremelanotide was only prescribed to 1% of FSIAD patients within 3 days of their initial diagnosis. Among patients with ED, 40% were prescribed a PDE5i and 2% received a referral.

In the multivariable logistic regression model of FSD patients, those diagnosed by a PCP had significantly lower odds of management compared to those diagnosed by an OB/GYN (aOR, 0.59; 95% CI, 0.51–0.69) (Table 2). In contrast, for patients with ED, diagnosis by a PCP had significantly higher odds of management (aOR, 1.52; 95% CI, 1.36–1.64) compared to diagnosis by a urologist. Patients diagnosed by an “other” clinician type had lower odds of both FSD and ED management compared to those diagnosed by a specialist. Compared to married patients, single patients had significantly lower odds of FSD management (aOR, 0.72; 95% CI, 0.61–0.85). For patients with ED, there was no association between marital status and odds of management.

**Table 2:** Adjusted Odds of Receiving Sexual Dysfunction Management Based on Patient Characteristics

	All female sexual dysfunction (*N* = 6540)	Genitourinary syndrome of menopause (*N* = 4302)	Non-GSM FSD (*N* = 2739)	Erectile dysfunction (*N* = 16,591)
	aOR (95% CI)*	aOR (95% CI)*	aOR (95% CI)*	aOR (95% CI)*
Age > 55	1.99 (1.74–2.28)	0.97 (0.81–1.15)	1.94 (1.54–2.45)	0.82 (0.76–0.90)
Race/ethnicity				
White	Reference	Reference	Reference	Reference
Black	1.06 (0.87–1.30)	1.03 (0.81–1.30)	1.02 (0.72–1.43)	1.05 (0.94–1.16)
Other	0.99 (0.82–1.18)	1.13 (0.91–1.40)	0.71 (0.51–1.00)	1.04 (0.93–1.16)
Insurance status				
Private	Reference	Reference	Reference	Reference
Medicare	0.89 (0.78–1.02)	0.84 (0.72–0.98)	0.96 (0.70–1.32)	0.71 (0.65–0.76)
Other	0.88 (0.73–1.06)	1.11 (0.87–1.42)	0.85 (0.64–1.14)	0.87 (0.78–0.98)
Marital status				
Married/domestic partner	Reference	Reference	Reference	Reference
Separated/divorced/widowed	0.94 (0.81–1.09)	0.93 (0.79–1.09)	0.82 (0.59–1.15)	1.04 (0.94–1.15)
Single	0.72 (0.61–0.85)	0.99 (0.80–1.23)	0.58 (0.43–0.77)	0.98 (0.89–1.08)
Unknown/other	1.39 (0.76–2.57)	1.38 (0.68–2.79)	1.01 (0.28–3.70)	0.79 (0.54–1.16)
Diagnosing specialty				
Specialist^†^	Reference	Reference	Reference	Reference
Primary care	0.59 (0.51–0.69)	0.56 (0.47–0.67)	0.73 (0.56–0.97)	1.52 (1.36–1.64)
Co-managed	1.26 (0.88–1.80)	1.04 (0.68–1.60)	1.66 (0.95–2.88)	0.93 (0.71–1.22)
Other specialty	0.33 (0.29–0.38)	0.34 (0.29–0.40)	0.35 (0.27–0.44)	0.55 (0.50–0.60)

^*^aOR (95% CI), adjusted odds ratio (95% confidence interval). Mixed-effects logistic regression analyses included all variables listed in the table as main effects, and primary care physician identifier as random intercepts

^†^Specialist: OB/GYN for female sexual dysfunction, urologist for erectile dysfunction

In the sensitivity analysis restricting data to the years 2020–2022, we found no meaningful differences in the frequency of management or estimated effect sizes. In the sensitivity analysis restricting the definition of ED management to PDE5i prescription, we found no meaningful differences in the estimated effect sizes.

### Rate of Sexual Dysfunction Management Among PCPs

A higher percentage of PCPs diagnosed ED during the study period (77%) than FSD (48%). Among these physicians, the median FSD management rate was 25% (IQR, 0–50%) and the median ED management rate was 52% (IQR, 33–75%). Forty percent of PCPs who made at least one FSD diagnosis never managed FSD. Fourteen percent of PCPs who made at least one ED diagnosis never managed ED. Among those PCPs who diagnosed both FSD and ED during the study period, there was a weak, but statistically significant, correlation between FSD and ED management rates (*r* = 0.24, *p* < 0.001). This suggests that managing ED was not strongly associated with managing FSD.

## DISCUSSION

In our study of patients seen by a PCP within a large, integrated health system, we found men were diagnosed and treated for ED at higher rates than women were for any type of sexual dysfunction. This is unlikely solely the result of lower sexual dysfunction rates among women. Women were more frequently diagnosed with FSD by a specialist, whereas men were more frequently diagnosed by a PCP. Over three-quarters of PCPs diagnosed ED at least once during the study period, whereas only half diagnosed FSD. FSD patients received management at lower rates than ED patients overall. In part, this was driven by the finding that PCPs were significantly less likely to order FSD management than OB/GYNs. In contrast, PCPs were significantly more likely to order ED management than urologists. Finally, we found a weak correlation between the rates of ED and FSD management by PCPs, indicating that physicians with a higher propensity to manage ED do not necessarily have a higher propensity to manage FSD.

Prior cross-sectional, nationally representative surveys estimate a much higher prevalence of FSD than we observed: 12% for disorders of desire, arousal, and orgasm^2^ and 13–21% for dyspareunia,^3,4^ yet we found only 4% of female patients were ever diagnosed with FSD. This discrepancy is likely due to the manner in which the data were ascertained—survey-based studies allow women to report sexual problems anonymously, whereas to receive a diagnosis of FSD, they would need to disclose these challenges to their physician. A qualitative study of PCPs and specialists experienced in treating FSD described clinicians as hesitant to “open Pandora’s Box” and discuss sexual dysfunction that they may not be able to adequately treat.^20^ The ED diagnosis rate we observed was similar to other studies using administrative data.^21,22^ Thus, the substantially lower rate of FSD diagnosis we found likely represents underdiagnosis, which may result from discomfort addressing sexual dysfunction by women and clinicians.^16,23^

Our finding that female patients diagnosed by PCPs were significantly less likely to receive appropriate management aligns with current literature. One survey of PCPs found that 90% had little confidence diagnosing hypoactive sexual desire disorder, 90% had never screened a patient for it, and 98% had never prescribed a medication for it.^15^ In recent guidelines, ISSWSH calls on PCPs to manage FSD, given the unique, longitudinal relationship they build with patients.^1^ In order to realize this, interventions may be needed to increase PCPs’ confidence in diagnosing and managing sexual dysfunction in women, particularly around prescribing newly approved medications. Indeed, fewer than one in five patients diagnosed with FSIAD in our study received a prescription for flibanserin when diagnosed (approved in 2015, prior to the study period), and only one in one hundred received a prescription for bremelanotide (approved in 2019, partway through the study period). These low prescription rates are likely driven at least in part by patient preferences. Flibanserin has a black box warning because it can cause syncope if taken soon after drinking alcohol.^24^ Some patients may opt against bremelanotide because it is an injection. Further, patients may have high out-of-pocket costs for both medications.

GSM was both the most commonly diagnosed form of FSD and twice as likely to be managed compared to with other FSD diagnoses. There are several possible explanations for this. GSM has physical exam findings that make it easier to identify and patients can raise GSM symptoms (i.e., vaginal dryness) without discussing sexual function. Additionally, clinicians are more familiar with the established and efficacious first-line treatment options for GSM (i.e., local or systemic estrogen replacement, and over-the-counter products).^25,26^ In contrast to clinician-reported discomfort with treatment of low desire, one survey of PCPs and specialists found that 76% reported having prescribed topical therapies for GSM.^25^

We found nearly half of ED patients received management, which is higher than some studies that defined management as filling a PDE5i prescription (22–25% treatment),^22,27^ but lower than another that also defined management as receiving a prescription (59% treatment).^7^ ED patients diagnosed by PCPs in our study were significantly more likely to receive management compared to those diagnosed by urologists, and this effect was maintained in our sensitivity analysis limiting the definition of management to PDE5i prescriptions only. In part, this may be because we defined ED management as a referral to urology or PDE5i prescription. One prior study found that compared to PCPs, urologists were more likely to prescribe non-PDE5i medications (i.e., intracavernosal injections) and see more complex patients for whom PDE5is may not be appropriate treatment.^7^ While PDE5is represent the majority of prescriptions provided by urologists,^7^ we may have underestimated ED management by urologists.

This study has limitations. We narrowly defined sexual dysfunction management as either a referral or prescription within 3 days of the initial sexual dysfunction diagnosis. We therefore were unable to account for some scenarios of appropriate management which would not have appeared in the chart. For instance, clinicians may recommend reading materials or use of over-the-counter products (i.e., vaginal lubricants/moisturizers) as first-line treatments for certain FSD diagnoses.^1^ This is an important consideration for GSM, as studies report lubricants to be equally efficacious as vaginal estrogen.^28,29^ Likewise, many women routinely see both a PCP and OB/GYN; PCPs may advise patients to discuss sexual problems with their OB/GYN without a referral. Additionally, specialists may provide procedural treatment of sexual dysfunction. We did not account for the role of patient preference in guiding management strategies. Patients may decline medication prescriptions due to high out-of-pocket costs or side effects. We anticipate this most frequently affects flibanserin and bremelanotide, which have much higher out-of-pocket costs compared to generic PDE5is. Further, we cannot confirm that medications were prescribed specifically for FSD, as medications in this analysis also treat other conditions. As we identified patients based on ICD codes in the EHR, we may have underestimated the frequency with which sexual dysfunction was discussed during visits. Lastly, this study included patients seen by PCPs at one healthcare system in Ohio, which may limit generalizability.

## CONCLUSION

Given the prevalence of FSD and availability of treatment options, discussion of sexual function at healthcare appointments is critical. We found FSD was diagnosed at a considerably lower rate based on EHR documentation compared to survey-based estimates, suggesting it is under-diagnosed. Although ISSWSH has identified PCPs as having a critical role in diagnosing and managing FSD,^1^ patients in our study who were first diagnosed by a PCP were less likely to have orders for management placed compared to those diagnosed by an OB/GYN. In contrast, ED patients diagnosed by a PCP were more likely to receive management than those diagnosed by urologists. Our work suggests that PCP-focused interventions may improve FSD treatment rates. However, further research about factors underlying patients’ preferences and key barriers faced by practitioners in providing treatment is needed to guide intervention development.

## Supplementary Information

Below is the link to the electronic supplementary material.

Supplementary file1 (DOCX 29 KB)

## Data Availability

Data may be available upon execution of a data use agreement with Cleveland Clinic.
