# Supplementary material for: Gap in Sexual Dysfunction Management Between Male and Female Patients Seen in Primary Care: An Observational Study
Source: J Gen Intern Med. 2024 Sep 4;40(4):847–53. doi: 10.1007/s11606-024-09004-1 (PMC11914646; doi:10.1007/s11606-024-09004-1)
Supplement: Supplementary file 1 — Supplementary file1 (DOCX 29 KB) [file 11606_2024_9004_MOESM1_ESM.docx]

**Appendix**

| Supplemental Table 1. Billing Codes Used to Identify Sexual Dysfunctions | | |
| --- | --- | --- |
|  | ICD-9 Codes | ICD-10 Codes |
| Female sexual interest/arousal disorder | 302.71, 302.72, 799.81 | F52.0, F52.1, F52.22, R68.82 |
| Female orgasm disorder | 302.73 | F52.31 |
| Genitopelvic pain/penetration dysfunction | 302.76, 306.51, 625.0, 625.1, 625.7 | F52.5, F52.6, N94.1, N94.2, N94.81 |
| Genitourinary Syndrome of Menopause | 627.3 | N95.2 |
| Other/unspecified female sexual dysfunction | 302.70, 302.79, V41.7 | F52.8, F52.9, R37 |
| Erectile Dysfunction | 607.84 | F52.21, N52 |
